# A New Model for PhD Elective Course

**DOI:** 10.12669/pjms.333.13259

**Published:** 2017

**Authors:** HR Ahmad, Satwat Hashmi, MH Mahmood

**Affiliations:** 1 HR Ahmad, Department of Biological and Biomedical Sciences Aga Khan University, Karachi, Pakistan; 2 Satwat Hashmi, Department of Biological and Biomedical Sciences Aga Khan University, Karachi, Pakistan; 3 MH Mahmood Department of Biological and Biomedical Sciences Aga Khan University, Karachi, Pakistan

An elective course for PhD students in cardiovascular and respiratory sciences was modeled and developed by a team of faculty at the Aga Khan University, Karachi. This course dealt with the multidisciplinary integrated concepts of heart, circulation and respiration components of human physiology. The understanding of basic science concepts enables students to better understand problems to derive solutions. Only those who understand how the healthy human body functions can recognize changes in diseased body to interpret the investigation rightly for an effective treatment. This forms the basis of dialectical imagination and experimentation.

This was a unique course of its kind where the concept of ‘lecture debate’ was introduced. Students and faculty indulged in mutual learning and difficult concepts were dealt with thought provoking visual cues e.g. elucidating data in the form of graphs and tables instead of textual slides.

The student’s feedback was encouraging. They remained highly motivated and committed throughout this course as being active team learners. They took active part in session discussions, execution of experimental work and tutorials. Their status of pre-knowledge enabled them to understand problems in light of application of currently grasped multidisciplinary integrated concepts. This offered a unique opportunity of turning teaching into learning mode.

## Content

Students at the end of the course were found to have developed the curiosity for learning and were able to think in response to challenges. This was evidenced by critical analysis of concepts through experimentation.

The following concepts of cardiovascular and respiratory system forms the content of this course while integrating morphology, biochemistry and physiology. This integration forms the basis of physiological sciences.

*Cardiac Mechanics
*Cardiac Cycle
*Cardiac Rhythm
*Cardiac Electricity
*Hemodynamics
*Micro-circulation
*CVS Control System
*Pulmonary Mechanics
*Gas Exchange
*Gas Transport
*Respiratory Control System

## Strategies

Cardiovascular and respiratory concepts were continuously developed in interactive learning sessions. This was supplemented with experimental lectures and tutorials. The course was of 12 weeks duration with two lectures, one tutorial and one laboratory work per week. This was supplemented with take home assignments of reading articles associated with the topics. The analysis of current scientific literature on the topics covered was critically debated in the context of premises and conclusion. This new model enabled the students to transform their imagination into experimentation to test the correlation between the two variables in order to arrive at an evidence based conclusion. Such a strategy of experimentation based learning could be possible through a horizontal enabling environment. Thus, this enabled to unfold the creative energy of students.

## Assessment

The assessment was based on the following distribution of marks. It included 30% Continuous assessment (CAT), 20% student presentation and 50% formal written examination. CAT was based on the students’ performance in the lecture debate and laboratory sessions. Student presentations during tutorial sessions were peer-reviewed by faculty involved in the module. Comprehensive written examination contained Best Choice and Extended Matching Questions and short integrated essay questions which included analysis and interpretations of data.

## Outcome

This course was a unique model of how active thinking driven by curiosity for knowledge enhanced the learning experience of students. How thinking oscillates between a group and an individual and how critique enhances learning. One is winged with the ability of going back and forth with imagination and experimentation to be enlightened and nurture the learning curve of consciousness.
